# Multi-omics insights into dietary zinc–mediated reprogramming of the gut–nasal ecosystem in allergic rhinitis

**DOI:** 10.3389/falgy.2026.1801629

**Published:** 2026-03-16

**Authors:** Shiqi Yan, Zhijuan Zhang, Chunyan Xia, Peng Liu, Junbai Ma, Jian Liu, Tong Wu, Hao Wang, Ruixia Ma

**Affiliations:** 1 The Second Clinical Medical College, the First People’s Hospital of Yinchuan, Ningxia Medical University, Yinchuan, China; 2 Department of Laboratory Medicine, the First People’s Hospital of Yinchuan, Yinchuan, China; 3 Ningxia Key Laboratory of Infection and Immunity, Department of Pathogenic Biology and Medical Immunology, School of Basic Medical Sciences, Ningxia Medical University, Yinchuan, China; 4 Otolaryngology Head and Neck Surgery Hospital, the First People’s Hospital of Yinchuan, the Second Clinical Medical College, Ningxia Medical University, Yinchuan, China; 5 School of Laboratory Medicine, Ningxia Medical University, Yinchuan, China

**Keywords:** allergic rhinitis, zinc, gut microbiota, gut-nasal axis, inflammation, short-chain fatty acids

## Abstract

**Introduction:**

Allergic rhinitis (AR) represents a prevalent kind of allergic disorders. Increasing evidence has revealed the critical role of gut microbiota dysbiosis in its onset and progression. The gut-nasal axis connecting the immune system and intestinal microecology offers a novel strategy for AR intervention.

**Methods:**

This study employed a multi-omics approach to systematically investigate the therapeutic effects and underlying mechanisms of dietary Zinc (Zn) supplementation at a series of concentrations on AR.

**Results:**

The results indicated that Zn may exert alleviated AR in rats, especially at a dose of 150 ppm. Mechanistically, we found the effectiveness of Zn may attributed to inhibiting inflammation, restoring gut microbiota balance, enhancing microbial metabolite-short-chain fatty acids (SCFAs) production, and regulating the nasal mucosa metabolism.

**Discussion:**

Collectively, our study indicate that dietary Zn intervention ameliorates AR in rats via gut-nasal axis, which may potentially provide a novel therapeutic treatment for the control of the disease.

## 1 Introduction

Allergic rhinitis (AR) is a common allergic condition that affects approximately 10%–20% of the global population (1), imposing a globally prevalent health issues and massive economic burden (2). For AR, allergens enter the body to excessively activate nasal mucosal cells including the activation of mast cells and basophils. The activation of these cells leads to the release of inflammatory mediators, resulting in vasodilation, increased glandular secretion, and typical symptoms such as nasal itching, clear rhinorrhea, and sneezing. The subsequent recruitment of inflammatory cells triggered a Th2-type immune response and delayed-phase reactions such as nasal congestion. The most common treatments include antihistamines, intranasal corticosteroids, and leukotriene receptor antagonists. However, long-term application carries certain side effects and limited efficacy. Bisides, biologics are expensive and their effectiveness varies among individuals. Thus, the pathogenesis of AR and its intervention strategies urgently require further investigation.

Zinc, an essential trace element in the human body, participates in tissue structure and catalytic regulation. Zinc deficiency can lead to a series of symptoms including loss of appetite, impaired immune function, and growth retardation (3). Zinc is deficiency in AR patients through large-scale surveys (2). Combining zinc supplementation with nasal corticosteroids alleviated nasal congestion, itching, and sneezing while reducing serum-specific IgE levels (4). Subsequent basic research revealed that varying zinc doses exert immunomodulatory effects on AR in mice via the p38MAPK pathway (5). While different zinc sulphate concentrations regulate the immune function of P815 mast cells through the ST2-p38 pathway (6). Thus, we propose that the efficacy of Zn treatment may be attributed to the modulation of immune function.

The gut microbiota is considered as the second largest genome in human, with up to 10^10^–10^11^ CFUs (7). The bacterial species encompass 50 genera and over 400 species (8). SCFAs are one of the crucial benefical metabolic products of bacteria, with 95% of which being absorbed in the colon, mainly including acetic acid (60%), propionic acid (25%), and butyric acid (15%) (8). SCFAs serve as an important kind of energy sources for the gut barrier, accounting for 60%–70% of the energy supply for colonic epithelial cells and 5%–15% of the total caloric intake in humans (9). Besides, their downstream receptor, GPR43 (also called FFAR2), is primarily expressed on immune cells and plays a role in suppressing inflammation (10). However, the exact impact of diverse concentrations of Zn on the progression of AR and underlying mechanisms by modulating gut/nasal microbiota and associated metabolites remain unclear.

Therefore, by employing a multi-omics approach, we elucidated how dietary Zn ameliorates AR via the gut-nasal axis. Variations in dietary Zn levels resulted in parallel changes in inflammatory responses and gut homeostasis. Collectively, this study may provide an experimental support for the translational potential of Zn as a dietary intervention candidate for AR.

## 2 Materials and methods

### 2.1 Animal experiments

A total of 48 five-week-old female Sprague–Dawley (SD) rats were obtained from the Laboratory Animal Centre of Ningxia Medical University and approved by the Ethics Committee of Ningxia Medical University (No.2024-180). Animals were raised in the Experimental Animal Center of Ningxia Medical University under pathogen-free (SPF) conditions. The room had a relative humidity of 60%, an ambient temperature of 25 ℃, and 12-hr light-dark cycles to maintain the normal circadian cycle.

**Figure 1:**
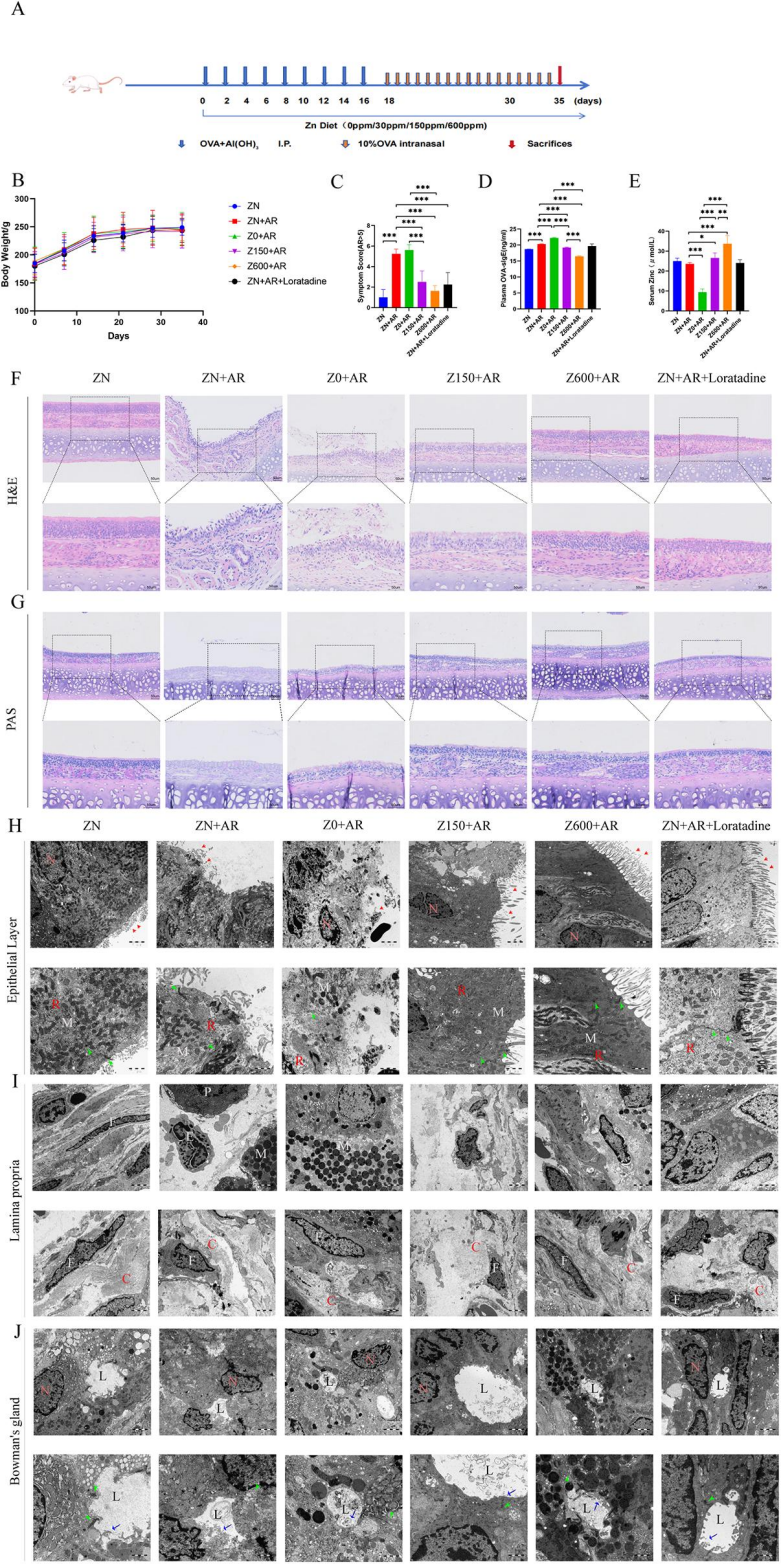
Zn improved OVA-induced allergic rhinitis. **(A)** An abridged general view of the experimental protocol AR rat modeling and Zn intervention process. **(B)** Changes in body weight (*n* = 8). **(C)** Nasal symptom scores. **(D)** Detection of OVA-IgE in rats Plasma using ELISA. **(E)** Zn concentrations in serum. **(F)** HE staining (100×, 200× magnification). **(G)** PAS staining(100x, 200× magnification). **(H)** Transmission electron micrograph of the nasal mucosal epithelial surface showing junctional complex (green arrow), cilia (red arrow), mitochondria (M), rough endoplasmic reticulum (R), and a regular nucleus (N). The first row displays a magnification of 10,000×. The second row shows a 20,000× magnification of the first row's section. **(I)** Electron micrograph of the lamina propria of the nasal mucosa showing collagen fibers (C) and fibroblasts (F), mast cell (M), eosinophil (E), and plasma cell (P). The first row is shown at 10,000× magnification. The second row presents the intrinsic layer and additional localized regions, also at 10,000× magnification. **(J)** Transmission electron micrograph of a Bowman's gland: nucleus (N), lumen (L), microvilli (blue arrow), and intercellular junction complex (green arrow). The first row is magnified at 10,000× magnification. The second row shows a close-up of the Bowman's gland, magnified at 20,000× magnification. *p < 0.05, **p < 0.01, ***p < 0.001.

The rats were randomly cate-gorized into six groups (8 rats/group): Zn-normal control group (ZN, 30 mgZn/4,000 kcal), AR model group (ZN + AR, 30 mg Zn/4,000 kcal), Zn-deficient diet-fed group (Z0 + AR, 0 mg Zn/4,000 kcal), high-Zn-fed group (Z150 + AR, 150 mg Zn/4,000 kcal), excess-Zn-fed group (Z600 + AR, 600 mg Zn/4,000 kcal), positive group (ZN + AR + Loratadine, 30 mg Zn/4,000 kcal, loratadine was administered orally by gavage at 1 mg/kg from day 0 to day 35). Diets containing altered Zn levels were synthesized by Jiangsu Synergy Bio (Nanjing, China). Zn was added as carbonate salts (11), using the AIN-93G standardized diet. Since Zn supplementation affected food intake, rats in each group were pair-fed to ensure isocaloric intake. The Zn-deficient group was allowed to eat freely, and the daily food intake of the Zn-deficient group from the previous day was used as the baseline to determine the feed quantities for the other groups. Except for the control group that received normal saline instead of Ovalbumin (OVA) throughout the experiment, all other groups underwent AR modeling. After an adaptation period of one week, the main experiment was started. Then, every other day (day 0, 2, 4, 6, 8, 10, 12, 14 and 16) they were given an intraperitoneal injection of 0.3 mg of OVA mixed with 3 mg of aluminium hydroxide powder dissolved in 1 mL of normal saline. After the basic sensitization, 50 μL of 10% OVA (12) solution was administered to each nasal cavity from day 18 to day 35 (Figure 1A).

Body weight of each rat in diverse group was weekly measured. At days 35 nasal symptom scores were assessed. Animals were placed into individual observation boxes and observed during 30 min, during which we counted the number of nose-rubbing events, sneeze events and nasal discharge events occurred (5). Next, blood samples were collected from the rats via the abdominal aorta under anesthesia. Plasma and serum were obtained through centrifugation of blood samples (600 × g, 10 min at 4 ℃) and then stored at −80 ℃. Nasal lavage fluid (NLF) was collected aseptically, centrifuged, and the pellet was stored at −80 ℃. Nasal and colonic mucosa samples were collected for istochemical analysis. Stool samples were also collected and immediately stored at −80 ℃ for further analysis.

### 2.2 Histopathological evaluation of nasal and colon

Nasal mucosal and colon tissues from rats were fixed in 4% paraformaldehyde for 48 h at room temperature, followed by gradient dehydration and embedding into paraffin wax blocks. The 4 μm sections were prepared for Hematoxylin and Eosin (H&E), Toluidine Blue (TB), and Periodic Acid–Schiff (PAS) staining. Tissue morphology were observed under microscope after staining with above-mentioned methods. The number of mast cells were counted using the ImageJ 1.8.0.322 software after staining with toluidine blue.

### 2.3 Zn determination

The measurement of Zn concentration was used with 0.5 mL of serum using an automatic biochemical analyzer (au5800; beckman coulter, USA).

### 2.4 Determination of plasma OVA-sIgE, IL-6, IL-10,IL-1β and TNF-α

Plasma was used to measure the expression levels of OVA-sIgE and inflammatory cytokines including IL-6, IL-10, IL-1β, TNF-α, according to the procedures provided by commercial enzyme-linked immunosorbent assay (ELISA) kits (Jingmei Biotechnology, China; Proteintech, China). The absorbance was measured at 450 nm with a microplate reader (Infinite® 200 PRO, Tecan, Switzerland).

### 2.5 Quantitative real-time PCR

Total ribonucleic acid (RNA) was isolated from frozen nasal mucosa tissue with a commercial RNA extraction kit (R6834, Omega, United States). Complementary DNA (cDNA) was synthesized via reverse transcription on the T100 Thermal Cycler system, and qPCR assays were subsequently performed on the qTOWER3/G 3107B system. GAPDH served as the reference gene for normalizing target gene expression. The used primers were listed below (Table 1). The relative expression was calculated using the 2^−ΔΔCT^ method. The results were expressed as the n-fold difference relative to the control group.

**Table 1:** Primers used for qRT-PCR.

Gene	Forward primer (5′ → 3′)	Reverse Primer (5′ → 3′)
GAPDH	GACATGCCGCCTGGAGAAAC	AGCCCAGGATGCCCTTTAGT
p65	CCCAGCCCTATGCCTTTTCA	AGGCCTGGTTTGAGATCTGC
GPR43	TCCTTGATCCTCACGGCCTA	CAGCAGCAACAACAGCAAGT
IL-1β	ATGCCTCGTGCTGTCTGACC	TTTGTCGTTGCTTGTCTCTCCTTG
TNF-α	GCCCAGACCCTCACACTCAG	CCGCTTGGTGGTTTGCTACG
IL-6	CTTCCAGCCAGTTGCCTTCTTG	TGGTCTGTTGTG GGTGGTATCC
IL-10	CTGCTATGTTGCCTGCTCTTACTG	GGG TCTGGCTGACTGGGAAG

### 2.6 Immunofluorescence analysis

To further evaluate the expression levels and localization of nasal mucosal pathway proteins and assess the integrity of the intestinal barrier, we used immunofluorescence to analyze differences among the experimental groups. As mentioned previously (13), the prepared 4μm nasal mucosal and colon sections were incubated with primary antibodies including anti-p65 (80979-1-RR, diluted 1:350, proteintech), anti-GPR43 (19952-1-AP, diluted 1:350, proteintech), anti-ZO-1 (ER41204, diluted 1:1,500, huabio), and anti-Occludin (ET1701-76, diluted 1:500, huabio), and then added with corresponding secondary IgG (SA00003-2, diluted 1:350, proteintech), subjected to immunofluorescence (IF) analysis. Images were acquired using a Apexview APX100 all-in-one microscope (Olympus, Japan) and a shengqiang SQS-120P Slide Scanner (shengqiang, China). Images were analyzed using Image J.

### 2.7 Immunohistochemistry (IHC)

To confirm the infiltration of inflammatory cells into the nasal mucosa, immunohistochemical staining with CD11b (CY5019, diluted 1:100, Abways) was used. Sections were deparaffinized with xylene and hydrated through a graded ethanol series. Subsequently, the sections were filled with EDTA antigen repair solution for 15 min in boiling water. Then, the slides were incubated in 3% H_2_O_2_ for 15 min and blocked with goat serum. The sections were incubated overnight at 4 ℃ with CD11b. After rinsing, the slides were incubated with secondary antibody for 1 h. After 5 min of reaction with the substrate-chromogenic agent, the sections were followed by counterstaining with hematoxylin. Image J software was used for statistical analysis. The image was obtained with a shengqiang SQS-120P Slide Scanner (Shengqiang, China).

### 2.8 Measurement of plasma LPS in rats

LPS concentrations in plasma samples were determined according to the manufacturer's instructions for the limulus amebocyte lysate kit (Xiamen Bioendo Technology Co., Ltd, Xiamen, China). The optical density value was detected at 545 nm.

### 2.9 The microbial communities in rat feces, and rat NLF were analyzed by 16S rRNA sequencing

Six rats from each group placed in sterile cages to collect fresh feces that were frozen in liquid nitrogen immediately after collection, and stored at −80 ℃ until DNA extraction. The nasal lavage fluid was processed and preserved as previously described (14, 15).

Fecal DNA was extracted from the samples using a commercial DNA extraction kit (DP328,TIANGEN BIOTECH CO., LT, Beijing), while the NLF microbial genomic was extracted using the CTAB method. The diluted genomic DNA was used as a template for PCR amplification. Depending on the selected sequencing region, specific barcoded primers were employed together with the Phusion® High-Fidelity PCR Master Mix (New England Biolabs) and a high-fidelity DNA polymerase to ensure amplification efficiency and accuracy. The forward primer 5′-AGAGTTTGATCCTGGCTCAG-3′ and reverse primer 5′-GNTACCTTGTT ACGACTT-3′ were used for amplification of the full-length V1–V9 region of 16S rRNA. Bacterial rRNA gene sequencing was performed using the PacBio platform (Pacific Biosciences of California,lnc). Original sequence data were deposited into NCBI SRA database with accession number PRJNA1373183.

### 2.10 Determination of fecal SCFAs in rats

The quantification analysis of fecal SCFAs was performed using a 1,300 gas chromatograph (Thermo Fisher Scientific, USA), coupled with ISQ 7000 mass spectrometric detector (Thermo Fisher Scientific, USA), equipped with an Agilent HP-INNOWAX (30 m × 0.25 mm ID × 0.25 μm) HP-5MS column (Suzhou Bionovogene Co., Ltd). Helium was used as the carrier gas (flow of 1 mL/min). The temperature program was: 90 ℃(initial), then 10 ℃/min to 120 ℃, 5 ℃/min to 150 ℃, and 25 ℃/min to 250 ℃ (hold for 2 min). Data analysis was performed using the R package Ropls.

### 2.11 Non-targeted metabolomics

An appropriate amount of nasal mucosa tissue was precisely weighed into a 2 mL centrifuge tube, and 1,000 µL of extraction solution was accurately added, followed by the addition of a stainless steel bead. The sample was homogenized in a tissue grinder at 50 Hz for 60 s, and the process was repeated twice. The homogenate was then sonicated for 15 min at room temperature, followed by a 15 min incubation on ice. The mixture was centrifuged at 12,000× g 4 ℃ for 10 min. The entire supernatant was then transferred to a clean centrifuge tube and evaporated to dryness. The dried extract was reconstituted with 400 µL of 50% acetonitrile containing 4 ppm 2-chloro-L-phenylalanine. Finally, the reconstituted solution was filtered, and the filtrate was transferred to sample vials for LC–MS analysis (16). Hromatographic Conditions: Chromatographic separation was performed on a Thermo Vanquish UHPLC system (Thermo Fisher Scientific, USA) equipped with an ACQUITY UPLC® HSS T3 column (2.1 × 100 mm, 1.8 µm; Waters, Milford, MA, USA). The flow rate was set at 0.3 mL/min, the column temperature was maintained at 40 ℃, and the injection volume was 5 µL. In positive ion mode, the mobile phases consisted of 0.1% formic acid in acetonitrile (B2) and 0.1% formic acid in water (A2). The gradient elution program was as follows: 0–1 min, 10% B2; 1–5 min, 10%–98% B2; 5–6.5 min, 98% B2; 6.5–6.6 min, 98%–10% B2; 6.6–8 min, 10% B2. In negative ion mode, the mobile phases consisted of acetonitrile (B3) and 5 mM ammonium formate in water (A3). The gradient elution program was as follows: 0–1 min, 10% B3; 1–5 min, 10%–98% B3; 5–6.5 min, 98% B3; 6.5–6.6 min, 98%–10% B3; 6.6–8 min, 10% B3 (17).

Mass spectrometry conditions: a Thermo Orbitrap Exploris 120 mass spectrometer (Thermo Fisher Scientific, USA) equipped with an electrospray ionization (ESI) source was used for detection, with data acquired in both positive and negative ion modes. The spray voltage was set to 3.50 kV in positive mode and −2.50 kV in negative mode. Sheath gas flow rate was 40 arb, and auxiliary gas flow rate was 10 arb. The capillary temperature was 325 ℃. Full-scan MS experiments were performed at a resolution of 60,000 over an m/z range of 100–1,000. For MS/MS analysis, the HCD (higher-energy collisional dissociation) mode was employed with a collision energy of 30% and a secondary resolution of 15,000. The top four most intense ions were selected for fragmentation, and dynamic exclusion was applied to eliminate redundant MS/MS acquisitions (18).

### 2.12 Transmission electron microscopy (TEM)

Nasal mucosa tissue specimens (approximately 1 × 1 × 3 mm^3^ in size) were immediately collected and immersed in 2.5% glutaraldehyde fixative and stored at 4 ℃. After rinsing with PBS three times, the specimens were fixed with osmium tetroxide for 1 h, followed by three washes with ultrapure water. Subsequently, the tissues underwent dehydration through a graded ethanol series (30%, 50%, 70%, 80%, 90% and absolute alcohol) for 15 min each. The specimens were sequentially incubated in mixtures of acetone and embedding resin at ratios of 3:1, 1:1, and 1:3 for 5 h each, followed by incubation in pure resin overnight. Thereafter, the pure resin was replaced twice, with each exchange lasting 2 h, after which the specimens were then incubated at 45 ℃ for 4 h and polymerized at 65 ℃ for 48 h. Ultrathin sections (60–80 nm) were cut using an ultramicrotome (Leica LKB-V) and stained with 2% uranyl acetate and lead citrate. Then, the samples were examined using a transmission electron microscope (H-7650, Hitachi, Japan), and images were captured for subsequent analysis (19).

### 2.13 Statistical analysis

Statistical analysis was performed using GraphPad Prism 9.4 (GraphPad Software Inc., La Jolla, CA, USA) and SPSS version 24.0 (IBM Inc., Armonk, NY, USA). The data were expressed as the mean ± SEM. Differences among groups were analyzed using one-way ANOVA. According to the normal distribution, the differences between the two groups were analyzed by the Student's *t*-test (2-tailed). For non-normally distributed data, the Mann–Whitney *U*-test is recommended. For correlation analysis, Spearman methods were performed. *p* < 0.05 was considered statistically significant.

## 3 Results

### 3.1 Supplementation with Zn attenuated OVA-induced AR in rats

There was no significant difference in body weight (BW) during initial and subsequent treatments (Figure 1B). Symptom scores were significantly increased in AR model group comared to control group (*p* < 0.001), whereas Zn-deficiency exacerbated this socres (*p* < 0.001). Importantly, diverse concentrations of Zn intervention alleviated this abnormal symptoms (Figure 1C). Additionally, compared to the control group, an increase in OVA-specific IgE was observed in AR model group (*p* < 0.001), while the Zn-deficient group showed an aggragated level (*p* < 0.001), whereas the Zn supplementation exhibited a notable alleviation in autoimmune-related IgE level (*p* < 0.001) (Figure 1D). Measurement of Zn levels in plasma found that it was lower in Zn-deficient group (*p* < 0.001), but dietary supplementation with low or high Zn doses exhibited an increase in plasma Zn concentration (*p* < 0.05) (Figure 1E). As shown in Figure 1F, HE staining indicated a mucosal epithelial damage in the model group, with thickened basement membrane, inflammatory cell infiltration in the lamina propria, and small vessel proliferation. Zn deficiency worsened nasal mucosal damage compared to the model group, showing partial loss of mucosal epithelium with inflammatory exudate, thickened basement membrane in the lamina propria, and extensive inflammatory cell infiltration with glandular destruction. Conversely, Zn supplementation attenuated these pathological changes, with mild basal lamina thickening and reduced inflammatory cell infiltration. Positive and control groups, exhibiting similar nasal mucosal features. PAS staining found that no goblet cells were observed in the nasal mucosa of the control group, whereas a large number of goblet cells were present in the model group. This phenomenon was exacerbated by Zn deficiency. However, the expression of goblet cells in the nasal mucosa was decreased in diverse Zn-supplemented groups (Figure 1G).

### 3.2 Zn alleviated the microscopic structure of the nasal mucosa in AR rats

In the control group, the nasal mucosal epithelial cells exhibited regular, with intact cilia surfaces, normal mitochondria, rough endoplasmic reticulum, and regular nuclei. The double-membrane structure of mitochondria remained intact. Cells were tightly connected via junctional complexes. Collagen in the lamina propria was neatly arranged, fibroblasts were largely normal, and no significant inflammatory cell infiltration was observed. In the model group, nasal mucosal epithelial cells exhibited partial ciliary loss, loose cytoplasm with vacuolation, and markedly fragmented nuclei (Figure 1H). The lamina propria of the model group showed infiltration by mast cells, plasma cell infiltration and eosinophil. Mitochondria exhibited vacuolar changes with swollen cristae. Collagen fibers were disorganized and sparse. Fibroblasts were irregularly shaped and shrunken (Figure 1I). In the Zn-deficient group, cilia were absent from the surface epithelium of the nasal mucosa. Cytoplasmic vacuolization was observed, with nuclei appearing regular round to oval in shape. Vacuolization of mitochondria and cristae swelling were observed in epithelial cells (Figure 1H). In the lamina propria, collagen fibers were markedly disorganized, accompanied by evident morphological deformation of fibroblasts, along with a significant increase in the number of mast cells (Figure 1I). In the high-Zn group, epithelial cells exhibited compaction with cilia loss improved compared to the mode group (Figure 1H). Nuclei were compacted, mast cell numbers were reduced, and cell junction complexes were blurred and damaged. Focal areas of collagen depletion, vascular congestion, and improved fibroblast morphology compared to the model group were also observed. In the excess-Zn group, the surface epithelium of the nasal mucosa was intact with preserved cilia (Figure 1H). Nuclear morphology, goblet cells, mitochondria, and rough endoplasmic reticulum (RER) appeared largely normal. Fibroblasts exhibited generally normal shapes, and collagen fibers were regularly arranged with increased quantity compared to the high-Zn group. Conversely, the positive group showed intact surface epithelium with preserved cilia, normal nuclei, and mitochondria (Figure 1H). The lamina propria of the nasal mucosa exhibited mild collagen thinning, with fibroblasts appearing largely normal (Figure 1I).

In the control group, Bowman's glands of the nasal mucosa exhibited normal nuclear morphology and uniformly electron-dense cytoplasm. Continuous microvilli lined the glandular lumen, and intercellular junctions remained intact (Figure 1J). For the model group, Bowman's glands displayed nuclear irregularities and cytoplasmic vacuolization. They were surrounded by irregular fibroblasts, and the microvilli within the lumen were noticeably reduced. In the Zn-deficient group, the extent of cytoplasmic vacuolization in Bowman's glands was greater than that observed in the model group, accompanied by a marked decrease in luminal microvilli. The high-Zn group showed Bowman's glands with regularly arranged nuclei in the surrounding cells. Compared with the model and Zn-deficient groups, these glands maintained intact intercellular junctions and demonstrated an increased number of microvilli in the lumen. In the excess-Zn group, Bowman's glands consisted of well-organized serous cells, with continuous microvilli lining the lumen and preserved intercellular junctions. Bowman's glands in the positive group contained glandular epithelial cells, and the lumen was lined with continuous microvilli while intercellular junctions remained intact.

### 3.3 Zn intervention suppressed plasma and nasal mucosal inflammation in AR rats

**Figure 2:**
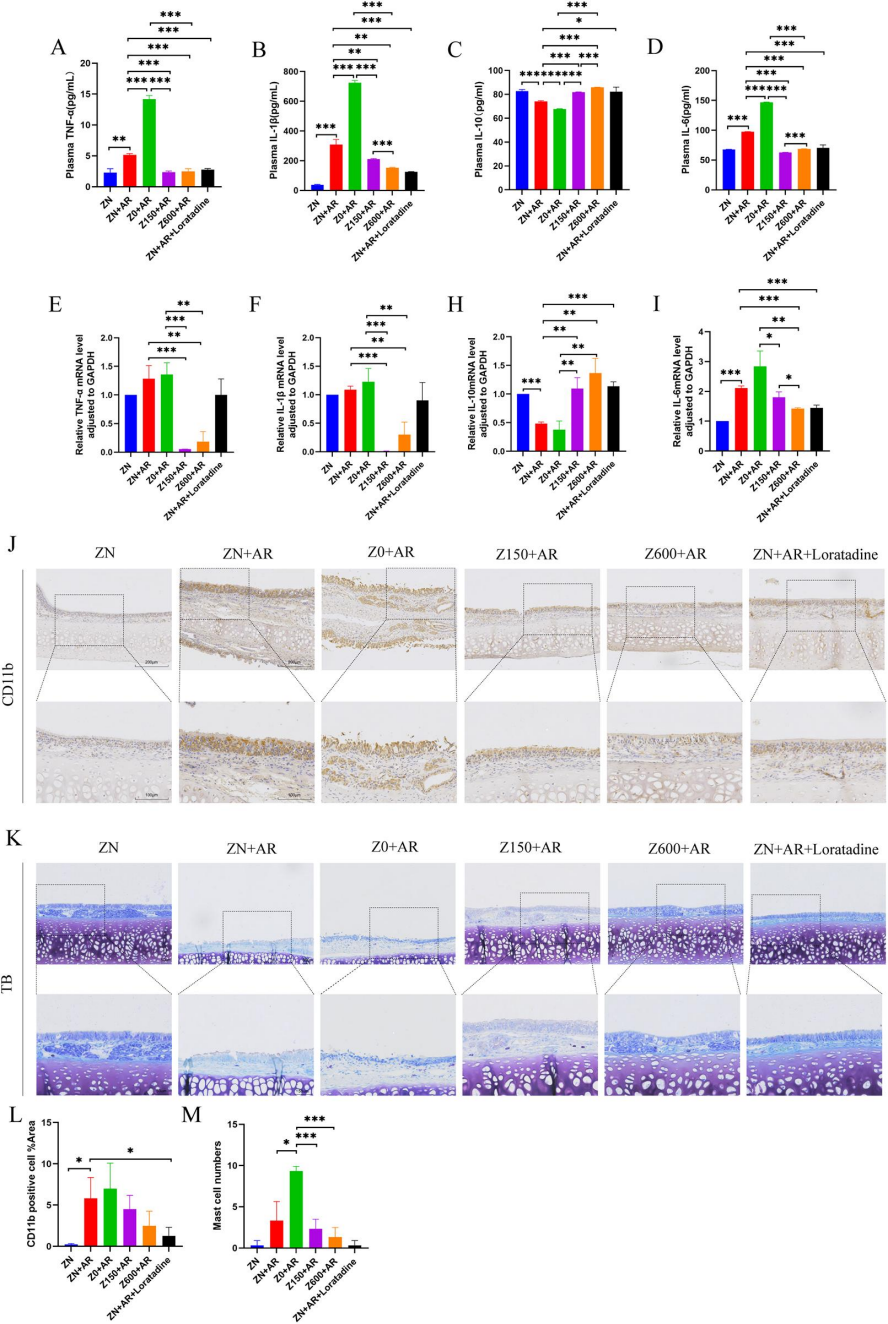
The effect of Zn on inflammatory factors in AR models. **(A–D)** The concentrations of TNF-α, IL-1β, IL-10, IL-6 in plasma of AR rats were measured by ELISA method. **(E–I)** The mRNA transcript levels of TNF-α, IL-1β, IL-10, IL-6 in the nasal mucosa of AR rats were detected using RT-qPCR. Zn supplementation reduces inflammatory infiltration and mast cell numbers in rat nasal mucosa and inhibits degranulation. The infiltration of myeloid-derived granulocytic inflammatory cells in thenasal mucosa of AR rats was determined by By immunohistochemistry **(J,L)**, while toluidine blue staining was employed to determine the number of mast cells and observe their degranulation status **(K,M)**. *p < 0.05, **p < 0.01, ***p < 0.001.

To further analyze the effects of Zn on systemic and nasal inflammation, we employed ELISA to measure inflammatory cytokine levels in the plasma and RT-qPCR to assess inflammatory impairment in the nasal mucosa. Following dietary supplementation with Zn, the concentrations of TNF-α, IL-1β, and IL-6 in the plasma and nasal mucosa of AR rats were significantly reduced, whereas IL-10 levels were elevated relative to the model group. Conversely, we found that plasma levels of pro-inflammatory TNF-α (*p* < 0.001), IL-1β (*p* < 0.001), and IL-6 (*p* < 0.001) were aggravated in the Zn-deficient group compared those in the AR model group, while anti-inflammatory IL-10 concentration was lower than in the model group (*p* < 0.001). We also measured *in situ* nasal mucosal TNF-α, IL-1β, IL-6, and IL-10, and found similar exacerbated pro-inflammatory cytokine levels in AR during Zn deficiency, while Zn supplementation exerted a beneficial effect (Figures 2A–I).

### 3.4 Zn modulated inflammatory cell infiltration in the nasal mucosa of AR rats

Immunohistochemical staining revealed CD11b expression in rat nasal mucosa (Figure 2J). Analysis of inflammatory cell infiltration in nasal mucosa showed that compared to the AR group, the Zn-deficient plus AR group exhibited severe nasal mucosal inflammation. Zn supplementation suppressed AR-induced reduction in CD11b expression and decreased its distribution in nasal epithelial tissue. CD11b level was significantly elevated in the model group, showing significant differences compared to the control and positive groups (Figure 2L, *p* < 0.05). Toluidine blue staining revealed mast cell infiltration and degranulation in the model group (Figures 2K,M). The Zn-deficient group exhibited worsened mast cell infiltration and degranulation compared to the model group (*p* < 0.05). Dietary supplementation with Zn reduced mast cell numbers with minimal degranulation. The control and positive groups had fewer mast cells with darker staining and no significant degranulation. Taken together, Zn modulates inflammatory cell infiltration, mast cell aggregation, and degranulation in the nasal mucosa.

### 3.5 Zn supplementation modulated the gut microbiota of AR rats

**Figure 3:**
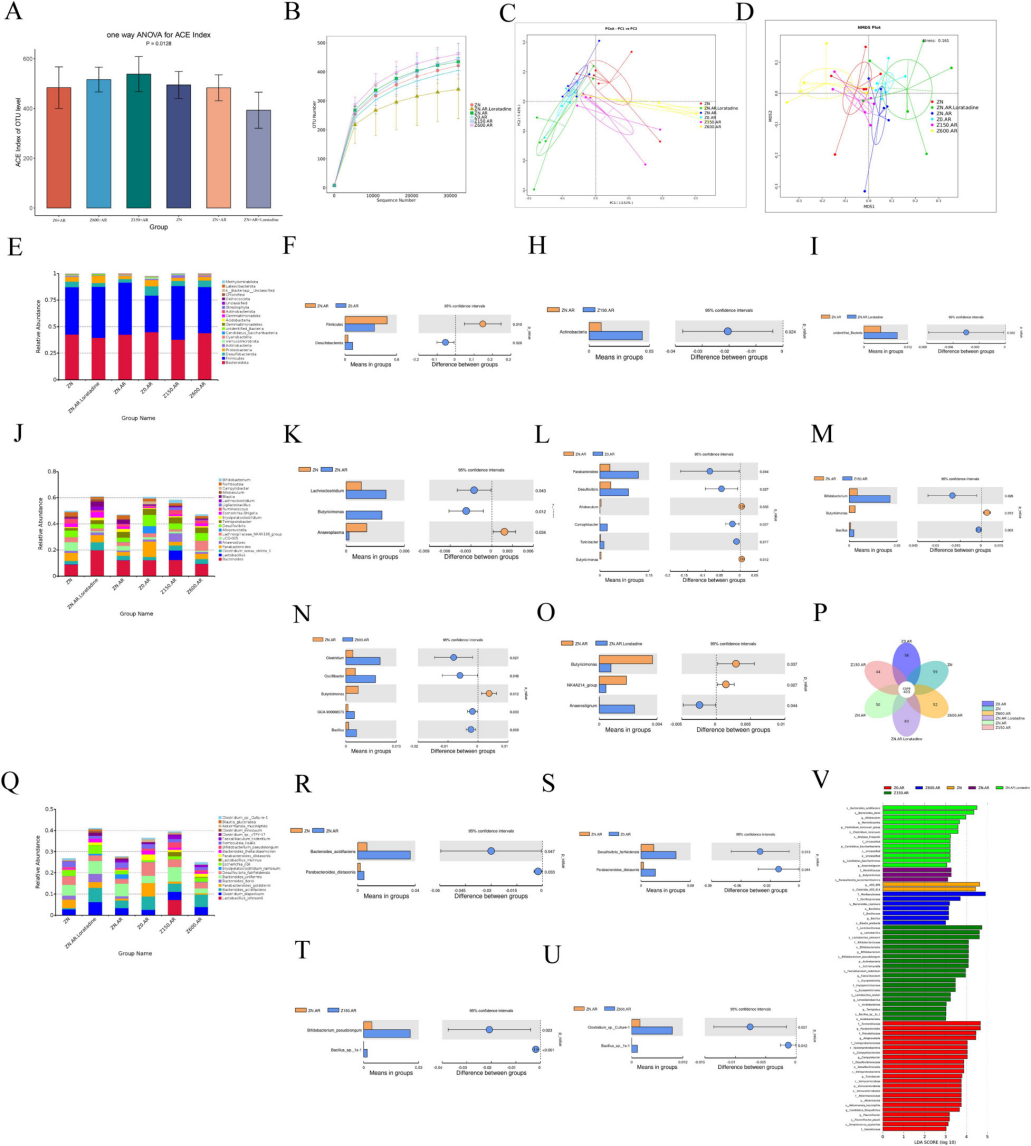
Zn modulated the composition of the gut microbiota in AR mice. **(A)** Ace index in each group. **(B)** Rarefaction Curve. **(C)** PCoA analysis. **(D)** NDMS analysis. **(E–I)** The phylum level. **(J–O)** The genus level. **(P)** Venn diagram. **(Q–U)** The species level. **(V)** The result of LEfSe analysis. Values are given as mean ± SEM.

To investigate changes in the gut microbiota, we performed 16S rRNA gene sequencing. Firstly, *α*-diversity analysis was employed to assess bacterial community abundance and diversity. ACE index analysis revealed significant changes in gut microbiota abundance and diversity under different Zn concentrations (*p* < 0.05) (Figure 3A). The curve flattens toward the end, indicated adequate sequencing coverage (Figure 3B). Analysis of bacterial community *β*-diversity using PCoA (Figure 3C) and NMDS (Figure 3D) revealed significant differences in microbial community structure among groups. Further analysis examined the gut bacterial composition of rats across different groups. Venn diagram (Figure 3P) showed 403 core species observed across all groups, with control, model, Zn-deficient, high-Zn, excess-Zn, and positive groups exhibiting 59, 50, 56, 44, 52, and 83 group-specific OTUs, respectively.

At the phylum level, *Firmicutes* and *Bacteroidetes* were dominant phyla across all groups. Compared to the model group, the Zn-deficient group exhibited a significantly reduced proportion of *Firmicutes* (*p* < 0.05) and an increase in *Desulfobacterota* (*p* < 0.05) (Figure 3F). Compared to the model group, the high-Zn group showed a significant increase in *Actinobacteria* (*p* < 0.05) (Figure 3H). Additionally, the positive group exhibited a significant increase in unidentified bacteria (Figure 3I).

Furthermore, LefSe was employed to identify essential differences in bacterial abundance (from phylum to species level) between the model group and other groups. For LefSe analysis, compared to the model group, at the genus level, the control group showed decreased *Lachnospiraceae* and increased *Anaeroplasma* (Figure 3K). *Butyricimonas* showed statistically significant elevation specifically in the model group, while it decreased in the remaining five groups (all *p* < 0.05). Zn-deficient group exhibited increased abundances of *Parabacteroides*, *Desulfovibrio*, *Campylobacter*, and *Turicibacter*, while *Allobaculum* abundance decreased (Figure 3L). The high-Zn group exhibited increased *Bifidobacterium* and *Lactobacillus* abundance (Figures 3M,V), while the excess-Zn group showed increased abundance of *Clostridium*, *Oscillibacter*, and *GCA-900066575* (Figure 3N). The positive group exhibited decreased abundance of *NK4A214* group and increased abundance of *Anaerostignum* (Figure 3O). *Turicibacter* exhibited changes across altered-Zn diets groups, increasing in 150 ppm Zn diet group while decreasing in Zn-deficient and 600 ppm Zn diet group (see Supplementary Figures S1A–C).

At the species level, *Parabacteroides_distasonis* increased in the model group (Figure 3R), with further increased abundance in the Zn-deficient group relative to model group (Figure 3S). Zn supplementation groups showed increased abundance of the genus *Bacillus* and species *Bacillus sp. 1s-1* (Figures 3T,U, Supplementary Figures S1D,E). Additionally, *Bacteroides_ acidilactis* and *Parabacteroides_distasonis* were increased in the model group. The Zn-deficient group further increased the abundance of *Desulfovibrio_fairfieldensis* and *Parabacteroides_distasonis* compared to the model group. Collectively, Zn supplementation increased beneficial gut bacteria relative to the model group for reshaing gut microbiota.

### 3.6 Zn intervention reshaped the microbiome in nasal irrigation solutions

**Figure 4:**
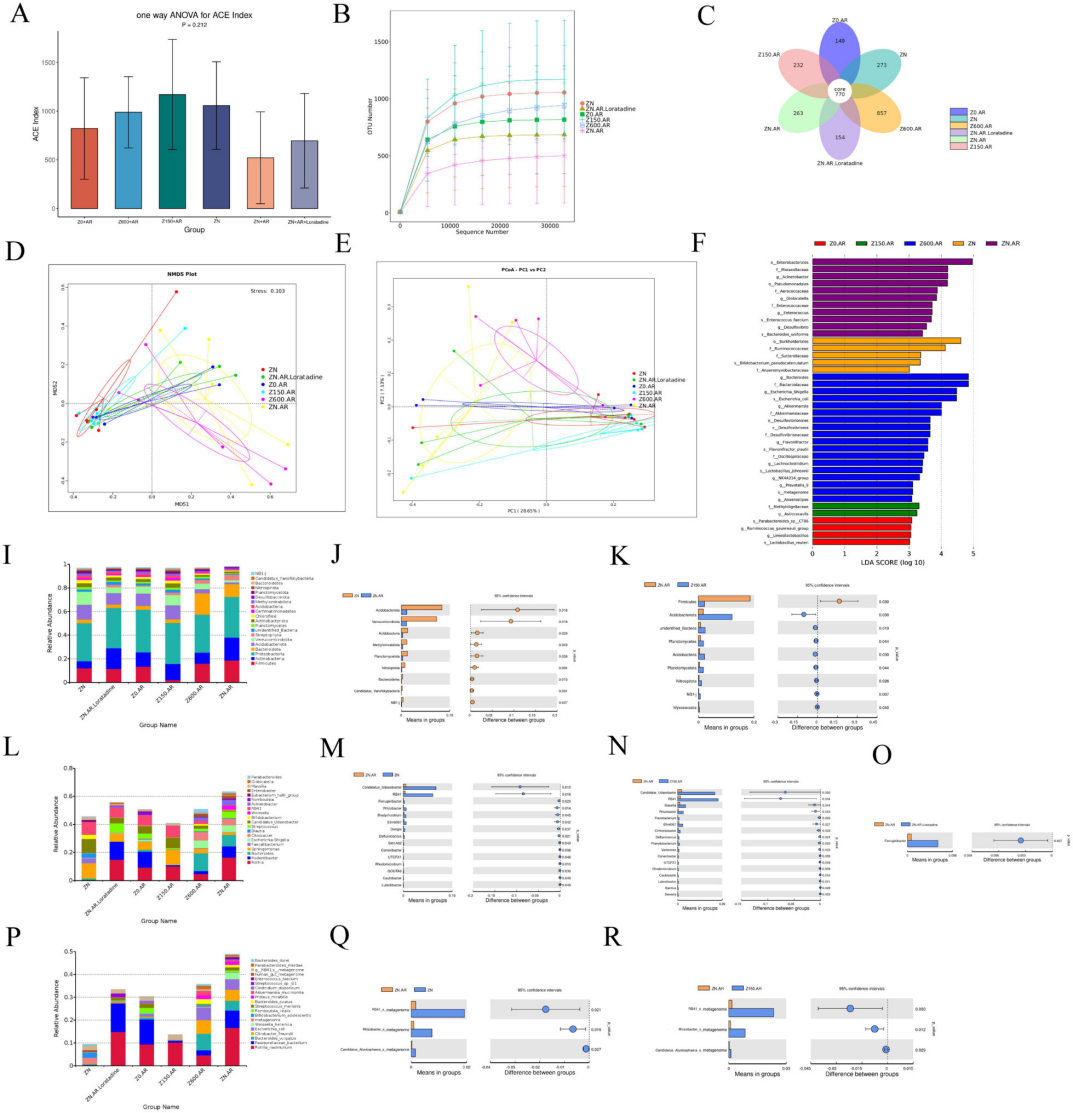
Zn modulated the composition of the nasal lavage fluid microbiota in AR rats. **(A)** Ace index in each group. **(B)** Rarefaction Curve. **(C)** flowerdata **(D)** NDMS analysis. **(E)** PCoA analysis. **(F)** The result of LEfSe analysis. **(I–K)** The phylum level. **(L–O)** The genus level. **(P–R)** The species level. Values are given as mea*n* ± SEM. *p < 0.05, **p < 0.01, ***p < 0.001.

Analysis of *α*-diversity was conducted to evaluate microbial community diversity and abundance. We found significant differences in the ACE index at the species level among nasal lavage fluid samples from different groups of rats (*p* = 0.00423) (Supplementary Figure S1F), whereas the ACE index at the operational taxonomic unit (OTU) level showed no significant difference (*p* > 0.05). Therefore, we concluded that differences exist in both species diversity and abundance within the microbial communities. Furthermore, microbiome composition shifted during Zn concentration interventions (Figure 4A). NMDS and PCoA analyses of *β*-diversity (Figures 4D,E) revealed significant differences in microbial community structure between the groups. The model group showed significant differences in microbiota composition compared to both the control group and the high-Zn group (*p* < 0.05). Furthermore, there was also a difference in microbiota composition between the two Zn supplementation groups. Further analysis of microbial composition across groups revealed 770 core species, as shown in the petal plot (Figure 4C). The numbers of species specific to the control, model, Zn-deficient, high-Zn, excess-Zn, and positive groups were 273, 263, 149, 232, 857, and 154, respectively.

Combined LEfSe analysis (LDA > 3, Figure 4F) and t-test were used for further analysis. At the phylum level, the three dominant phyla in the nasal lavage fluid across all groups were *Proteobacteria, Firmicutes* and *Actinobacteriota*, followed by *Bacteroidetes* and *Acidobacteria* (Figure 4I). Compared to the model group, both the control and high-Zn groups showed increased abundances of *Acidobacteriota*, *Acidobacteria*, *NB1-j*, *Planctomycetota*, and *Nitrospirota* (Figures 4J,K). Specifically, the control group exhibited elevated abundances of *Verrucomicrobiota*, *Methylomirabilota*, *Bacteroidetes*, *Candidatus*_*Yanofskybacteria* (all *p* < 0.05). In contrast, 150 ppm Zn supplementation resulted in a decreased abundance of *Firmicutes*, while the abundances of *unidentified_Bacteria*, *Planctomycetota*, and *Myxococcota* were increased (Figures 4I–K). Indeed, these bacterial groups primarily exist in environmental niches (20, 21).

At the genus level, the model group had higher abundances of *Acinetobacter*, *Globicatella*, *Enterococcus*, and *Desulfovibrio*. Compared to the model group, the control and 150 ppm Zn-supplemented groups exhibited higher abundances of *Candidatus_Udaeobacter* and *RB41*. Each group also possessed uniquely enriched genera: *Asticcacaulis* was elevated in the high-Zn group, while *Ruminococcus*, *Bacteroides*, *Akkermansia*, *NK4A214_group*, *Anaerostipes*, and *Subdoligranulum* were more abundant in the excess-Zn diet group.

At the species level, *Enterococcus_faecium* and *Bacteroides_uniformis* were more abundant in the model group compared to the others. The control group specifically showed an increased abundance of *Bifidobacterium_pseudocatenulatum*. Zn supplementation and deficiency altered the nasal microbiota at the species level in AR rats: *Lactobacillus_johnsonii* was higher in the excess-Zn group, while *Lactobacillus_reuteri* was more abundant in the Zn-deficient group. Collectively, the analysis of nasal lavage fluid microbiota indicates that Zn modulates the nasal microbiota in AR rats.

### 3.7 Zn supplementation significantly increased fecal SCFAs concentrations

**Figure 5:**
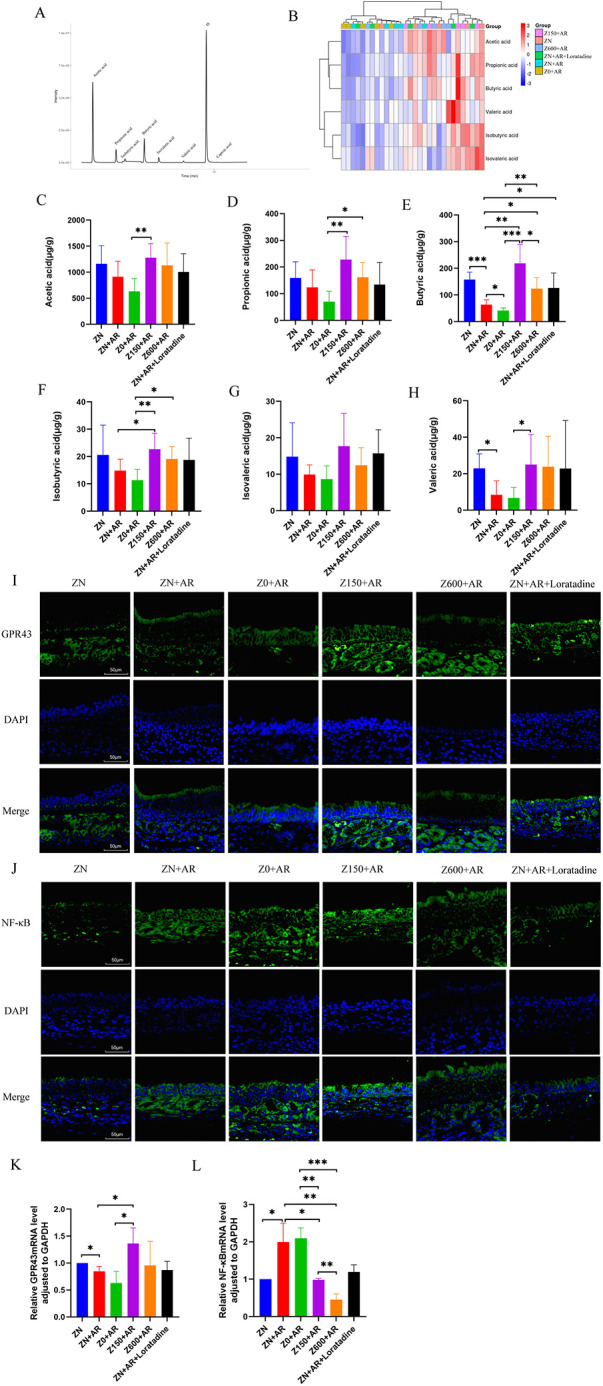
Zn modulated the intestinal contents of SCFA in AR rats. **(A)** Feces sample chromatogram. **(B)** Heat map clustering. **(C)** Acetic acid. **(D)** Propionic acid. **(E)** Butyric acid. **(F)** Isobutyric acid. **(G)** Isovaleric acid. **(H)** Valeric acid. Data were expressed as mean ± SD. Zn Intervention increased the expressions of GPR43 and reduced the number of NF-*κ*B in nasal mucosa tissue. **(I,J)** Representative IF profiles of GPR43, NF-*κ*B nasal mucosa tissue at 400× magnification. The mRNA levels of GPR43 **(K)**, NF-*κ*B **(L)** in nasal mucosa tissue. Data were expressed as mean ± SD. *p < 0.05, **p < 0.01, ***p < 0.001.

SCFAs mediate communication between the gut microbiota and the host immune system, maintain intestinal integrity and immune homeostasis, and regulate the balance between pro-inflammatory and anti-inflammatory cytokines in AR (22). Therefore, GC-MS assay was employed to detect SCFAs in rat feces, primarily including acetate, propionate, butyrate, isobutyrate, valerate, and isovalerate. The TIC chromatogram revealed that each of SCFA could be clearly distinguished and exhibited distinct peak shapes, indicating the reliability of the method and data (Figure 5A). The cluster heatmap displayed differences in SCFAs levels among different groups (Figure 5B). Compared to the model group, the high-Zn group exhibited the most pronounced increase across all groups, with statistically significant elevations in butyrate and isobutyrate (*p* < 0.05) (Figures 5C–H). Zn-deficient group showed a notable decrease in butyrate (*p* < 0.05). The excess-Zn group demonstrated an increase relative to the model group but remained lower than the high-Zn group.

### 3.8 Zn intervention suppressed the inflammation by regulating GPR43-NF-κB signalling pathway in rat nasal mucosa

SCFAs interacts with GPR43 to inhibit the NF-κB signaling pathway in immune cells (23). Accordingly, the expression levels of NF-κB and GPR43 were observed in rat nasal mucosa (Figures 5I–L). We found increased fluorescence intensity of NF-κB (p65) in the AR group. In p65 + DAPI images, p65 was primarily expressed in cell nuclei, with higher expression in the Zn-deficient group compared to the model group (Figure 5J). Conversely, p65 expression levels were decreased in the high-Zn, excess-Zn, control, and positive groups compared with the model group, whereas Zn deficiency worsened this inflammatory gene expression (Figure 5J). Moreover, Zn supplementation increased GPR43 expression. RT-qPCR analysis revealed that Zn supplementation statistically significantly inhibited the OVA-induced upregulation of p65 expression in nasal mucosal tissue (Figure 5L). Zn supplementation significantly reduced p65 mRNA expression in the nasal mucosa (*p* < 0.05). Compared to the model group, GPR43 in nasal mucosal tissue (Figure 5K) showed increased in the 150 ppm Zn diet group (*p* < 0.05), with co-expression in the cytoplasm (Figure 5I). Further, Zn supplementation significantly reversed the OVA-induced upregulation of GPR43 expression and downregulated p65 expression in the nasal mucosal (Figure 5K).

### 3.9 Metabolomic analysis of rat nasal mucosa revealed alterations in metabolites regulated by dietary Zn

**Figure 6:**
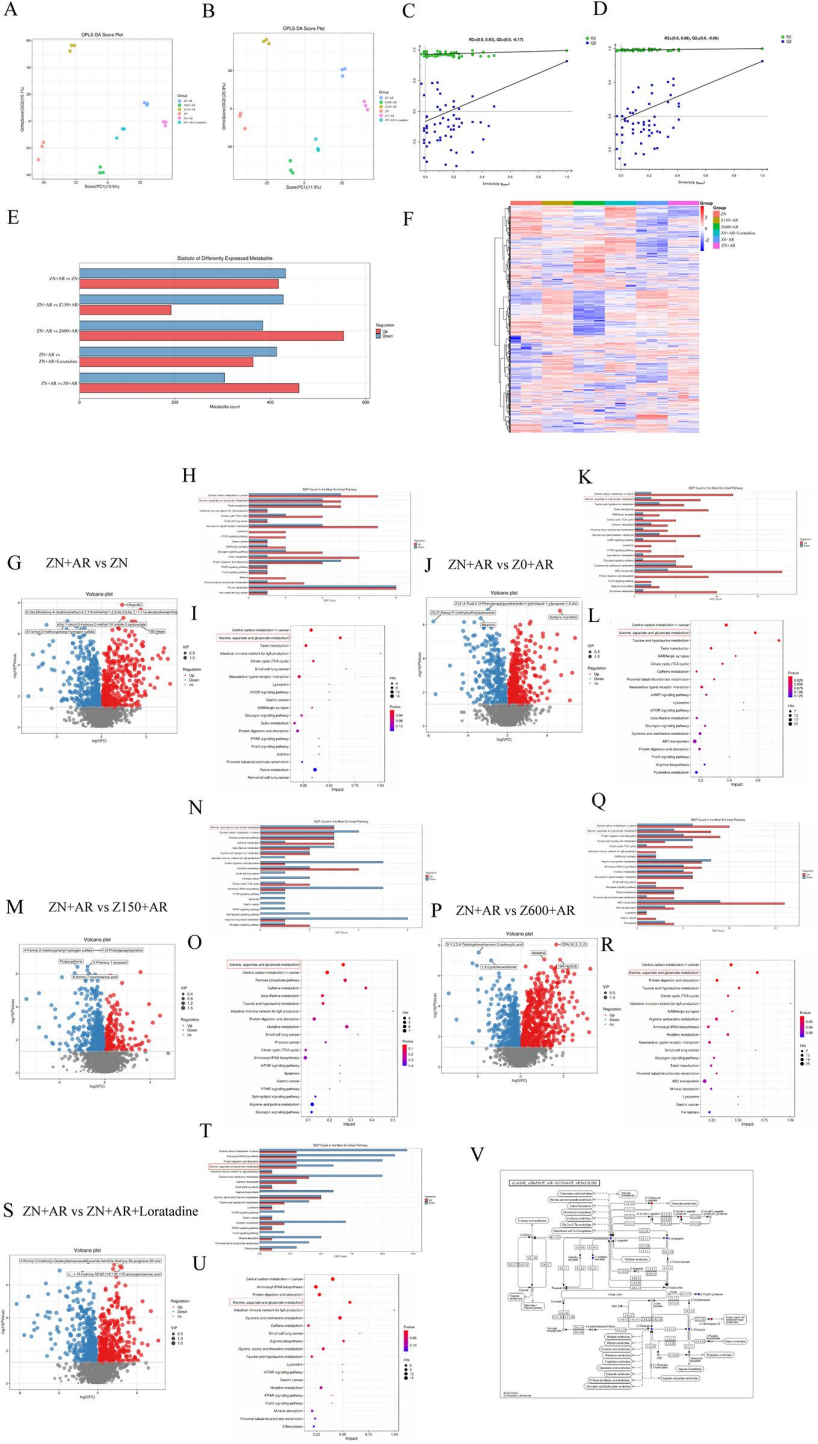
Zn regulated metabolic profiling of nasal mucosa tissue in the AR rats. Analysis separately in positive and negative ion modes. **(A,B)** OPLS-DA Score Plot. **(C,D)** The OPLS-DA permutation test diagram. **(E)** The number of differential metabolites between each two groups. **(F)** Heatmap of the different metabolites (DEMs). Volcano Plot of Differential Metabolites, the bar chart of the number of specific metabolites change in the pathways, bubble plots presented the differential metabolism pathways, between the model and control group **(G–I)**, between the model and Zn-deficient group **(J–L)**, between the model and high-Zn group **(M–O)**, between model and excess-Zn group **(P–R)**, or between the model and positive group **(S–U)**. **(V)** Amino Acid Metabolism-Alanine, Aspartate and Glutamate metabolism.

Positive and negative ion mode OPLS-DA analysis revealed distinct separation in metabolite distributions across the six groups (Figures 6A,B). Both R^2^ and Q^2^ values in the OPLS-DA displacement plot were lower than the original R^2^ and Q^2^ points in the upper right quadrant, indicating model reliability (Figures 6C,D). Using *p* < 0.05 and VIP > 1 as screening criteria, combined with the differential metabolite bar chart (Figure 6E) and nasal mucosa metabolite differential heatmap (Figure 6F), the number of differentially expressed metabolites between each group and the model group were 849, 764, 619, 938, and 777, respectively, compared to the control, Zn-deficient, high-Zn, excess-Zn, and positive groups. Metabolites differing between groups were shown in pairwise volcano plots (Figures 6G–M,P,S).

KEGG analysis was then performed on the differentially expressed metabolites, assigning them to distinct pathways. Overall, the top enriched metabolic pathway across all six groups was mineral absorption (*p* = 0.028040), followed by asthma (*p* = 0.057339) (Supplementary Figures S1G,H). This aligned with our two primary research objectives, confirming the validity of our model.

Analysis of metabolic pathways between the model and control groups (Figures 6H,I) revealed the top five pathways: carbohydrate metabolism [Central carbon metabolism in cancer, Citrate cycle (TCA cycle)], amino acid metabolism (Alanine, aspartate and glutamate metabolism), Neurobiology and Cellular Signaling (Taste transduction), and Immunological Pathway (Intestinal immune network for IgA production). The top 5 metabolic pathways between the model and Zn-deficient groups (Figures 6K,L) included carbohydrate metabolism (Central carbon metabolism in cancer), amino acid metabolism (Alanine, aspartate and glutamate metabolism, Taurine and hypotaurine metabolism), Neurobiology and Cellular Signaling (Taste transduction), and Nervous system (GABAergic synapse). Top 5 metabolic pathways between the model and high-Zn groups (Figures 6N,O) included carbohydrate metabolism (central carbon metabolism in cancer, pentose phosphate pathway), amino acid metabolism (alanine, aspartate and glutamate metabolism; beta-alanine metabolism), and Xenobiotic Metabolism (caffeine metabolism). The top 5 metabolic pathways between the model and excess-Zn groups (Figures 6Q,R) were carbohydrate metabolism [central carbon metabolism in cancer, citrate cycle (TCA cycle)], amino acid metabolism (alanine, aspartate and glutamate metabolism; taurine and hypotaurine metabolism), Digestive system (protein digestion and absorption). The top 5 metabolic pathways between the model and positive groups (Figures 6T,U) included carbohydrate metabolism (central carbon metabolism in cancer), amino acid metabolism (alanine, aspartate and glutamate metabolism), genetic information metabolism (aminoacyl-tRNA biosynthesis), digestive system (protein digestion and absorption), immunological pathway (intestinal immune network for IgA production). Alanine, aspartate, and glutamate metabolism (Figure 6V) frequently appeared among the top five pathways enriched in the model and several other KEGG pathway groups, suggesting that different intervention groups may regulate this pathway. Compared with the model group, the number of metabolites involved in the alanine, aspartate and glutamate metabolism pathway was decreased in the control group, decreased in the Zn-deficient group, unchanged in the high-Zn group, decreased in the excess-Zn group, and increased in the positive control group.

### 3.10 Zn supplementation enhanced gut integrity of the intestinal barrier

**Figure 7:**
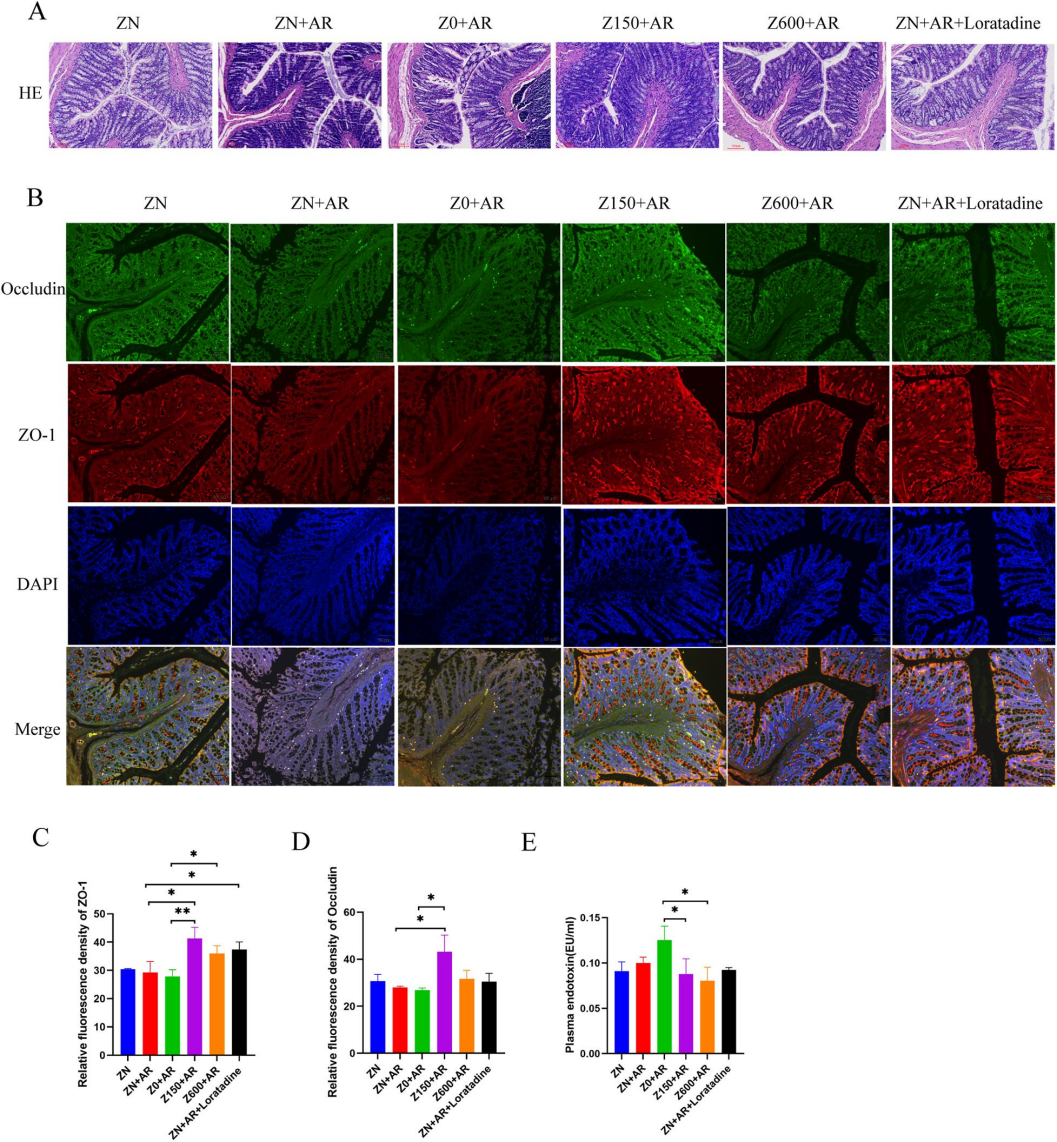
Zn significantly changed the expression of ZO-1 and occludin in the colon of AR rats. **(A)** Colonic tissue morphology was observed by HE staining. **(B)** ZO-1 and Occludin expression in the colon of AR rats was determined by immunofluorescence staining. Semi-quantitative analysis of the relative levels of ZO-1 **(C)** and Occludin **(D)** by densitometric analysis. Data were expressed as mean ± SD. **(E)** The level of LPS in plasma. *p < 0.05, **P < 0.01, ***p < 0.001, ba*r* = 100 µm (HE).

H&E staining revealed intestinal pathological alterations (Figure 7A). Rats in the model group exhibited colonic epithelial necrosis and sloughing, glandular disorganization, and visible inflammatory infiltration. The Zn-deficient group showed more severe manifestations than the model group, with exacerbated glandular disorganization. Compared to the model group, the control and positive groups presented near-normal appearances, showing no significant inflammatory infiltration and regular glandular structures. Symptoms improved after supplementation with different concentrations of Zn. Studies on the rat colonic barrier revealed that, compared to the model group, Zn deficiency combined with model exacerbated intestinal barrier damage. Zn supplementation inhibited the AR-induced reduction in tight junction protein expression (ZO-1 and Occludin) and decreased the distribution of intestinal mucosal epithelial cells (Figure 7B). Moreover, compared to the model group, the expressions of protein ZO-1 and Occludin were elevated in the 150 ppm Zn diet group (Figures 7C,D, *p* < 0.05). Zn deficiency exacerbated intestinal barrier damage in AR rats, whereas Zn supplementation appeared to suppress inflammation, restore terminal villus structure, and improve the intestinal mucosal barrier. Therefore, we conclude that Zn modulates intestinal barrier integrity.

### 3.11 Dietary Zn reduced lipopolysaccharide (LPS) level in plasma

LPS derived from Gram-negative bacteria in the gut primarily translocates into the bloodstream through a compromised intestinal barrier, migrate to the nasal mucosa, trigger nasal inflammation, and exacerbate the progression of AR (24–26). Therefore, we further determined the effect of Zn supplementation on plasma LPS level in AR rats. Plasma LPS was elevated in the AR model group compared to the control group, with a more pronounced increase in the Zn-deficient AR model, although this difference was not statistically significant. Following Zn supplementation, LPS levels in the high-Zn and excess-Zn group were significantly lower than those in the Zn-deficient group (*p* < 0.05, Figure 7E). The above results suggested that Zn alleviated endotoxemia and improved intestinal barrier integrity, while reducing intestinal permeability.

### 3.12 Precise prediction of Zn content changed in AR rats through microbial community analysis

Spearman correlation analysis was performed to examine the relationship between gut microbiota phyla, genera, and serum Zn levels (Supplementary Figures S1I-Q). At the phylum level, serum Zn levels positively correlated with the relative abundance of *Candidatus_Saccharibacteria*. Receiver operating characteristic (ROC) analysis indicated that *Candidatus_Saccharibacteria* levels could be used for highly accurate discrimination of Zn status (AUC, 0.8611; Supplementary Figures S1J,K). At the genus level, *Turicibacter* exhibited a negative correlation with Zn concentration, being more abundant in the Zn-deficient diet group than that in the excess-Zn group. *Turicibacter* demonstrated good diagnostic accuracy in distinguishing Zn deficiency from Zn excess (AUC, 0.8889 and 0.8056, Supplementary Figures S1M,N). A species-level bacterium, *Flavonifractor_plautii*, was additionally identified as specific to low and excessive Zn levels (Supplementary Figure S1P,Q). Based on Spearman correlation analysis and ROC curves, *Flavonifractor_plautii* showed a negative correlation with serum Zn levels (*r* = −0.4852, *p* = 0.0163) and ROC curve AUC values ranging from 0.6806 to 0.8056 (Supplementary Figures S1P,Q).

### 3.13 Association between Zn-regulated gut microbiota and intestinal metabolites in AR immunity

**Figure 8:**
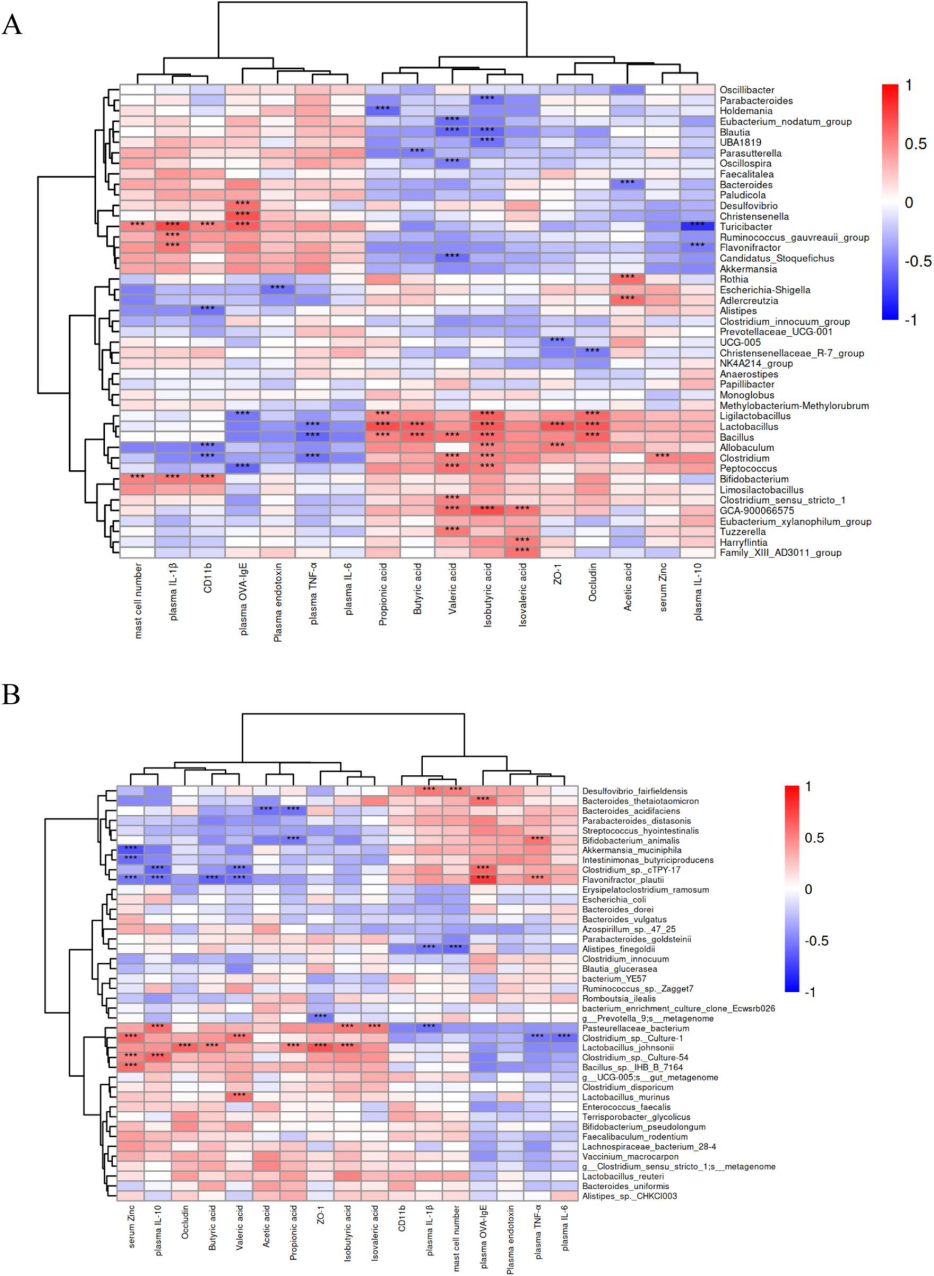
Correlation analysis between gut microbiota and inflammatory cytokines, tight junction proteins, inflammatory cells, and gut metabolites: at both the genus **(A)** and species **(B)** levels. Data are expressed as mean ± standard deviation. *p < 0.05, **p < 0.01, ***p < 0.001. Red and blue indicate positive or negative correlations, respectively.

Spearman correlation analysis was used to evaluate the associations between gut microbiota and cytokines, immune cells, SCFAs, and related parameters. At the genus level (Figure 8A), *Clostridium* showed a positive correlation with Zn concentration and negative correlations with CD11b-expressing cells and TNF-α. *Desulfovibrio*, *Christensenella*, and *Turicibacter* were positively correlated with OVA-sIgE. Among them, *Turicibacter* was negatively correlated with serum Zn levels and plasma IL-10 concentrations. *Ligilactobacillus*, *Lactobacillus*, and *Bacillus* were positively correlated with tight junction proteins such as ZO-1 or Occludin, as well as with propionic or butyrate. *Peptococcus* was negatively correlated with OVA-sIgE.

At the species level (Figure 8B), *Akkermansia_muciniphila*, *Intestinimonas _butyriciproducens*, and *Flavonifractor_plautii* were negatively correlated with serum Zn levels. *Flavonifractor_plautii* was negatively correlated with IL-10 and butyrate, but positively correlated with OVA-sIgE concentration. *Clostridium_sp._Culture-1*, *Clostridium_sp._Culture-54*, and *Bacillus_sp._IHB_B_7164* were positively correlated with serum Zn concentration. In addition, *Clostridium_sp._Culture-1* was negatively correlated with TNF-α and IL-6. *Lactobacillus_johnsonii* was positively correlated with butyrate and propionate and was also positively correlated with ZO-1 or Occludin. Collectively, the results suggested that Zn possess the ability to change the immunity of AR through the gut microbiota mediated pathway.

## 4 Discussion

Accumulating evidences have demonstrated that dysbiosis of the gut microbiota is closely associated with the occurrence and progression of allergic diseases (27). Changes in the compositions of gut microbiota may notably modulate the production of their metabolites, such as SCFAs, ultimately affecting numerous critical pathways related to energy homeostasis, nutrient absorption, and immune balance (28). In the present study, we found that Zn alters gut microbiota and metabolites in rats, regulating AR inflammation via the gut-nasal axis.

Gut micobiota dysbiosis is thought to play a crucial role in the pathogenesis of AR (27). In our experiment, we found that dietary Zn modification notably altered bacterial composition at the phylum, genus, and species levels in AR rats. Firstly, at the phylum level, *Firmicutes* and *Bacteroidota* were the dominant taxa across all six groups, consistent with previous findings (22, 29). Compared to the model group, the Zn-deficient group showed a significant decrease in *Firmicutes* and a marked increase in *Desulfobacterota*. *Desulfobacterota*, a Gram-negative pathogenic taxon, can produce endotoxins such as LPS and trigger inflammatory responses (30). In line with literature, *Firmicutes* was also reduced in the gut microbiota of prenatal Zn-deficient rats (31). At the genus level, *Turicibacter* exhibited specificity: its abundance was higher in the Zn-deficient group compared to the model group, and it showed a negative correlation with Zn content in AR rats. *Turicibacter* is currently regarded as a controversial bacterial genus. an increase in *Turicibacter* was reported in the heat-sensitive moxibustion (HM) group as a probiotic (32). In contrast, another study found that *Turicibacter* was enriched in the gut microbiota of patients with eosinophilic chronic rhinosinusitis with nasal polyps (eCRSwNP) and was positively correlated with the percentage of tissue eosinophils (33). In addition, a separate study showed that supplementation of dogs with Zn sulphate or Zn sulphate combined with enzyme addition significantly increased three gut bacterial taxa, including *Turicibacter* (34), which is inconsistent with our findings. This discrepancy may be related to differences in species and disease conditions. In the Zn-deficient group, the genera *Parabacteroides*, *Desulfovibrio*, and *Campylobacter* were enriched relative to the model group. These taxa are closely linked to inflammation (35–37), suggesting that aggravated inflammation in the Zn-deficient group may be associated with shifts in the gut microbiota. Conversely, butyrate-producing *Allobaculum* and *Butyricimonas* (38, 39), were reduced. In the high-Zn group, *Butyricimonas* and *Bifidobacterium* were also significantly increased. *Bifidobacterium* is involved in SCFA production (40), and its enrichment, along with other probiotics, may partly explain the higher SCFA levels in high-Zn group. Additionally, *Clostridium* increased in the excess-Zn group is enriched in healthy dogs without digestive disorders (41) and may be beneficial. At the species level, compared to the model group, the Zn-deficient group exhibited increased abundances of *Desulfovibrio_fairfieldensis* and *Parabacteroides_distasonis*, both of which has been reported to be associated with infection and disease (35, 42). The high-Zn group exhibited elevated *Bifidobacterium_pseudolongum*, a major *Bifidobacterial* species in mammals that can lower colonic 5-HT levels in mice (43). In addition, LEfSe analysis showed that *Lactobacillus_johnsonii* was also enriched in the high-Zn group. a*L. johnsonii* is a typical intestinal probiotic and possesses several beneficial properties, including anti-inflammatory activity, immunomodulatory effects, maintenance of gut microbiota balance, and protection of the intestinal barrier (44). In summary, Zn deficiency increased pathogenic bacteria in AR rats, while Zn supplementation promoted beneficial taxa. The impact of Zn on gut microbiota may involve several mechanisms: (1) bacterial competition for Zn via ZnuABC transporters, affecting colonization and growth under Zn-limiting conditions; (2) SCFAs inhibiting the growth of certain *Proteobacteria*, with antimicrobial efficacy varying with pH—e.g., weakening against *E. coli* when pH rises from 5 to 7 (45)—whereas Zn deficiency lowers intestinal pH and SCFA levels, further shaping microbial composition (46); (3) Zn absorption influenced by its chemical form: Zn carbonate in feed reacts with gastric acid to form zinc chloride, which is absorbed in the intestine; Zn ions may also interact with or antagonize the absorption of other metals and nutrients, limiting uptake of both Zn and competing elements. These factors collectively may explain how Zn modulates the gut microbiota, though more comprehensive mechanisms await further investigation.

Zn exhibited a regulatory effect on the levels of fecal SCFAs in rats with AR. Zn deficiency resulted in a marked reduction in SCFAs, whereas Zn supplementation significantly increased the levels of the immunomodulatory SCFAs acetate, propionate, and butyrate, with the most pronounced increase observed in the high-Zn group. Alterations in gut microbiota composition and associated metabolic pathways influence carbohydrate and glycan metabolism, as well as intestinal mucin degradation, thereby affecting the production of SCFAs (47). Beneficial microbial metabolites, such as SCFAs, are closely associated with reduced inflammatory markers and interactions with the host immune system (48). Therefore, we analyzed the inflammatory status in AR rats next.

Consistently, our study found that Zn deficiency aggravated symptoms and inflammation in AR rats, whereas dietary Zn supplementation significantly ameliorated these indicators. In this experiment, compared to the model group, the Zn-deficient group exhibited aggravated sneezing and nasal rubbing symptoms, along with damage to the mucosal epithelium, goblet cell hyperplasia, and inflammatory cell infiltration. However, Zn supplementation was able to reverse the above symptoms and histological changes. These indicators are commonly used to evaluate the therapeutic effects of drugs on AR (49, 50). In addition, plasma levels of OVA-sIgE and pro-inflammatory cytokines (TNF-α, IL-6, and IL-1β) were elevated, whereas the anti-inflammatory cytokine IL-10 was reduced in the Zn-deficient group; these alterations were corrected in the Zn-supplemented groups. This phenomenon was further confirmed at the mRNA transcription level in the nasal mucosa. These inflammatory factors (5, 22, 51) not only participate in the pathological allergic inflammatory response of AR but also have the potential to recruit and activate other immune cells, or to affect the structure of vascular endothelial cells, epithelial cells, and nerve cells, leading to tissue damage and remodeling. They can also stimulate the airway mucosa, airway epithelium, and smooth muscle to produce additional inflammatory mediators (52). The experimental results indicate that Zn supplementation suppresses systemic cytokine levels and local mucosal inflammatory responses in AR rats, suggesting that the trace element Zn plays an important role in immune regulation. Previous clinical studies support this conclusion, showing that Zn deficiency leads to impaired immunity and increased susceptibility to infections, whereas Zn supplementation enhances immune function and reduces susceptibility to infections (53–55). However, the precise mechanisms by which Zn exerts its effects in the body remain to be further elucidated in future studies.

The composition of microbial communities within the body is generally similar, but their relative abundances vary across different habitual anatomical sites, where they interact and influence one another. An imbalance in the gut microbiota can affect the relative abundance of respiratory tract microbiota. The microbiota of the respiratory tract plays a significant role in shaping the host's immune status (56). Further research on nasal microbiota revealed that Zn supplementation altered the nasal microbiota in AR rats. Inter-group community dissimilarity analysis demonstrated that the model group exhibited significant differences from the control group and the 150 ppm Zn-supplemented group, respectively. Significant differences were also observed between the two Zn-nutraceutical groups. Changes in nasal microbiota were observed in AR group rats, consistent with clinical AR studies, though the specific microbial shifts differed, possibly due to variations in research species and environments (57). The dominant phyla in nasal lavage fluid across groups were *Proteobacteria*, *Actinobacteriota,* and *Firmicutes*, aligning with findings in the literature (57). LEfSe analysis indicated a greater diversity of probiotics in the excess-Zn group at both the genus and species levels, such as the genus *Akkermansia* and the species *Lactobacillus_reuteri* and *Lactobacillus_johnsoni*. *Lactobacilli* are enriched in the nasal passages of healthy individuals, with certain species demonstrating anti-pathogenic effects by inhibiting the growth of upper respiratory pathogens *in vitro* and reducing inflammatory responses in airway epithelial cells when co-cultured with pathogens (58)*.* Similarly, enrichment of *Bifidobacterium_pseudocatenulatum* in the control group suggests a potential correlation between probiotic enrichment in the respiratory tract and allergic diseases. Although *Lactobacillus_reuteri* was enriched in the Zn-deficient group, the range of beneficial bacteria was limited, and the specific mechanisms remain unclear. The model group showed enrichment of non-probiotic bacteria, such as the genera *Desulfovibrio* and *Acinetobacter*, and the species *Enterococcus_faecium*. *Desulfovibrio* can both promote host health and contribute to disease onset and progression, effects closely linked to its metabolites (e.g., H_2_S and SCFAs) and biofilms (59). *Acinetobacter* abundance is also increased in AR populations (57), consistent with existing literature. *Enterococcus* exhibits dual characteristics: it can colonize the nasal cavity and is associated with drug resistance (60), while specific strains of *Enterococcus_faecium* may serve as probiotics for AR treatment (61). Overall, Zn supplementation altered the nasal microbiota in AR rats and increased the abundance of beneficial bacteria. The specific mechanisms require further investigation.

LPS, released into the bloodstream as a bacterial endotoxin, can help link the gut microbiota, intestinal barrier function, and systemic inflammation (13). Our study found that Zn concentration altered plasma LPS levels in AR rats. LPS concentrations in AR rats were significantly decreased by Zn, in comparison with the Zn-deficient group. Most gut microbiota belong to Gram-negative bacteria, whose cell walls are primarily composed of LPS. LPS level is considered one of the diagnostic markers of inflammatory diseases. After LPS is shed from the bacterial cell wall, it can enter the bloodstream through a damaged intestinal barrier (62). In the circulation, lipopolysaccharide-binding protein (LBP) detects LPS and facilitates its recognition by Toll-like receptors (TLRs) on macrophages or neutrophils, thereby inducing the secretion of pro-inflammatory cytokines such as IL-6 and triggering inflammatory responses (63). These inflammatory cytokines can activate the NF-*κ*B pathway, which has long been regarded as a canonical pro-inflammatory signaling pathway. NF-*κ*B exerts pro-inflammatory effects by regulating the expression of cytokines, chemokines, and adhesion molecules (64), thereby initiating a series of downstream cascade events. Therefore, we conducted subsequent investigations focusing on pathway-related molecular mechanisms.

The present study revealed that Zn may suppress the inflammation by modulating the expression of NF-κB and GPR43 in the nasal mucosa of AR rats. Under conditions of Zn deprivation, NF-κB expression was significantly upregulated, and supplementation with Zn markedly attenuated its expression, indicating that Zn played a pivotal role in mediating the anti-inflammatory effects of the intervention. Conversely, GPR43 expression decreased in the model and Zn-deficient groups but increased in the Zn-supplemented groups, the control group, and the positive control group, with the highest level observed in the high-Zn group—consistent with the elevation in SCFA levels. A potential mechanism is that SCFAs bind to SCFA receptors, inhibiting LPS-induced activation of NF-*κ*B in inflammatory cells and thereby suppressing inflammatory responses (63). On the other hand, SCFAs inhibit histone deacetylases, regulating the function and quantity of Th1 and Treg cells as well as modulating the levels of myeloid cell precursors, thereby influencing allergic diseases (65). Existing literature also indicates that butyrate supplementation can inhibit the p38 MAPK/NF-*κ*B signaling pathway in Tfh13 cells through interaction with the GPR43 (23), which aligns with the findings of this study. Specifically, Zn supplementation elevates SCFAs, activates GPR43, and inhibits inflammatory responses by suppressing LPS and NF-*κ*B activity. The downstream effects induced by pathway activation were further investigated through subsequent experimental studies.

Zn regulated non-targeted metabolites in the nasal mucosa. Screening revealed that the potential metabolic pathway marker for Zn is the Alanine, aspartate and glutamate metabolism pathway. Across all groups, this pathway was consistently identified as one of the top enriched metabolic pathways in KEGG analyses, and its enrichment was modulated by Zn concentration. This pathway has also been validated in other experiments. In a clinical study involving patients with Artemisia pollen-induced AR undergoing subcutaneous immunotherapy, patients were grouped into ineffective and effective responders based on treatment outcome. Post-treatment serum metabolomic analysis revealed four differential pathways, among which the Alanine, aspartate and glutamate metabolism pathway was consistent with our findings. Metabolites in these pathways are considered potential biomarkers for effective subcutaneous immunotherapy (66). Furthermore, similar results were obtained in mouse experiments. An AR model was established using CD169 gene-knockout mice and wild-type C57 mice, with non-AR modeled groups serving as controls, totaling four animal groups. Nasal lavage fluid was collected for non-targeted metabolomic sequencing. Results showed a high proportion of Organic acids and derivatives, and enrichment analysis ultimately identified the Alanine, aspartate and glutamate metabolism pathway, which was significantly downregulated in the two AR experimental groups (67). Additionally, bioinformatics analysis and plasma non-targeted sequencing have also linked this pathway to asthma (68, 69). This indicates that the Alanine, aspartate and glutamate metabolism pathway plays an important role in allergic diseases, which is consistent with our research. Furthermore, our study found that Zn can regulate this metabolic pathway. These metabolic changes may trigger a series of downstream effects, warranting further investigation.

We found that Zn supplementation promoted recovery of the intestinal mechanical barrier. The intestinal barrier is a dynamic system influenced by the composition of the gut microbiota and the activity of intercellular junctions (62). Both the present study and previous reports indicate that Zn intake alters the composition of the gut microbiota and the production of its metabolic products, SCFAs (11). Intestinal dysbiosis often results in increased intestinal permeability and impaired mucus-associated defenses, thereby increasing susceptibility to disease (62). Tight junctions (TJs) between intestinal epithelial cells are a critical component of the intestinal mechanical barrier. TJs consist of structural proteins and functional proteins; the main structural protein is Occludin, which forms the structural framework of TJs, while the principal functional protein is ZO-1 (49). In this study, the elevatted expression levels of ZO-1 and Occludin in the high-Zn group was consistented with our SCFA results. SCFA can regulate inflammatory responses by enhancing the function of tight junction proteins (TJPs), thereby increasing intestinal barrier integrity and preventing antigens from passing through the paracellular space (70). Inflammatory substances and signals such as LPS are transmitted throughout the body via the impaired intestinal barrier, triggering immune responses (49, 62). Thereby, we further investigated nasal mucosal inflammatory cells (CD11b-expressing cells) and the primary effector cells of AR (mast cells).

In addition, we found Zn supplementation reduced the expression of CD11b^+^ cells and also decreased mast cell count, while Zn deficiency aggravated the infiltration of corresponding inflammatory cells. CD11b is a protein subunit expressed on integrins on the surface of inflammatory cells (71). It is expressed on myeloid cells such as macrophages, neutrophils, dendritic cells, natural killer cells (72), eosinophils (73), and basophils (74), and may play an important role in inflammatory cell adhesion and activation. Detecting inflammatory cells helps to provide a more comprehensive understanding of the pathological process of allergic diseases. Mast cells are involved in allergic mucosal reactions (75). During pollen season, mast cells increase in number and patients' symptoms worsen, showing a positive correlation between the two (76). The cross-linking of IgE on mast cell membranes and the subsequent release of mast cell mediators (degranulation) are considered primary mechanisms associated with AR (77). In our study, an increase of mast cells with lighter staining in the model group, whereas the control group exhibited fewer mast cells with darker staining, suggesting the anti-inflammatory effect of Zn supplementation may contribute to the reduction of inflammatory infiltration with mast cells. Activated mast cells can release various bioactive mediators, such as histamine, prostaglandins, and leukotrienes, which can cause tissue edema and increased leakage (78). Previous studies have found that treatment with mugwort pollen and Zn sulphate significantly reduced the levels of ST2 and p38 in mast cells, and also alleviated the downregulation of Th2 cytokines (6), mitigating allergic inflammatory responses. These findings are consistent with earlier *in vitro* experiments demonstrating Zn-mediated regulation of inflammatory responses (6). To further analyze the changes induced by Zn, we conducted an in-depth investigation into the ultrastructure of the nasal mucosa in AR rats under Zn intervention.

Our study found that Zn modulated ultrastructural alterations in the nasal mucosa of AR rats. In the model group, partial loss of cilia, marked proliferation of damaged organelles, disruption of junctional complexes, inflammatory cell infiltration, morphological changes in fibroblasts, and disorganization or loss of collagen fibers were observed, which is consistent with previous reports (12). These pathological changes were more severe in the Zn-deficient group. Zn supplementation was able to reverse these ultrastructural abnormalities, and these findings were in agreement with the macroscopic pathological observations (HE, PAS, and TB staining) as well as the results of inflammatory marker analyses. Ultrastructural analysis of the nasal mucosa in patients with AR revealed capillary dilation, enhanced endothelial cell activity, and a large number of pinocytotic vesicles. Activated plasma cells with prominent endoplasmic reticulum were observed in the mucosa, while mast cells exhibited characteristic granule thickening and partial vacuolization (79). These findings are consistent with the ultrastructural observations in the nasal mucosa of AR rats, thereby confirming the successful establishment of the model.

Correlation analysis indicated that Zn interacted between the gut microbiota and the nasal mucosa via the “gut-nasal axis”. In AR rats, Zn induced changes in the gut microbiota. At the phylum level, we identified *Candidatus_Saccharibacteria*, an extremely widespread microbial group found in soil, sediments, wastewater, animals, and various clinical environments (80). The genus *Turicibacter* can serve as a marker for Zn concentration, as previously discussed and not elaborated further here. Correlation analysis revealed that *Ligilactobacillus*, *Lactobacillus*, and *Bacillus* are positively correlated with tight junction proteins such as ZO-1 or Occludin, as well as with propionate or butyrate. These genera include many probiotic species (81, 82). At the species level, *Flavonifractor_plautii* showed a negative correlation with serum Zn levels. It also exhibited negative correlations with IL-10 and butyrate, while showing a positive correlation with OVA-sIgE concentration. *Flavonifractor_plautii* has a dual nature, as a strict anaerobe, it can produce deaminotyrosine (DAT) (83) and elevate phytosphingosine levels to improve metabolic disorders (84), but it can also cause infections (85). *Lactobacillus_johnsonii* was positively correlated with butyrate and propionate, as well as with ZO-1 or Occludin. The positive correlation between *Lactobacillus_johnsonii* and intestinal tight junction proteins—a noteworthy finding—has been validated in the literature, confirming that this strain improves intestinal barrier function in pigs (86). Furthermore, *Akkermansia_muciniphila* showed a negative correlation with Zn concentration. In the literature, the correlation between *Akkermansia* and Zn depends on the duration of intervention (11). In the gastrointestinal tract, *Akkermansia* is the only cultured representative of *Verrucomicrobiota*. *Akkermansia_muciniphila,* the most well-characterized representative of this genus, is a mucin-degrading bacterium (87). By disrupting host mucus homeostasis, *A. muciniphila* exacerbated intestinal inflammation (88). Taken together, we have demontrated that the gut microbiota is associated with serum Zn levels and showed connections with inflammatory factors and cells, indirectly indicating that dietary Zn ameliorated inflammatory responses in AR rats by modulating gut microbes and metabolites, intestinal barrier function, and the immune system.

## 5 Conclusion

Our study revealed that dietary Zn supplementation ameliorates AR via the gut-nasal Axis through the multi-omics approaches. Mechanistically, Zn binds to GPR43, thereby inhibiting the NF-*κ*B signaling pathway, reducing inflammatory responses, and restoring the balance of the gut-nasal microbiota. Collectively, these findings highlight Zn as a promising therapeutic candidate for AR management. Nevertheless, further investigations are required to delineate its detailed molecular mechanisms and support its translation into clinical practice.

## Data Availability

The datasets presented in this study can be found in online repositories. The names of the repository/repositories and accession number(s) can be found in the article/Supplementary Material.
