# Supplementary figures and images for: Multi-omics insights into dietary zinc–mediated reprogramming of the gut–nasal ecosystem in allergic rhinitis
Source: Front Allergy. 2026 Mar 16;7:1801629. doi: 10.3389/falgy.2026.1801629 (PMC13033786; doi:10.3389/falgy.2026.1801629)

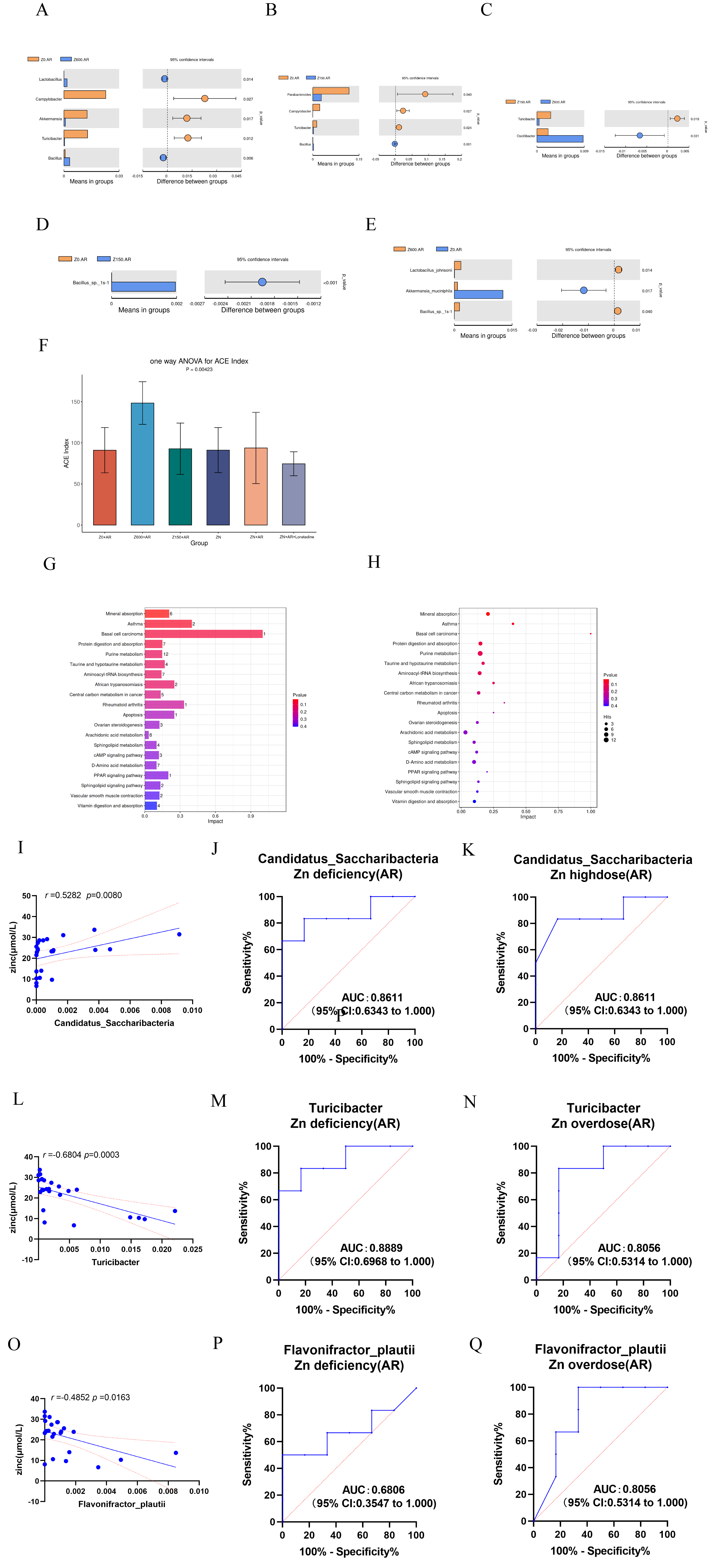

Supplement: Supplementary file 1 [file Image1.tif]

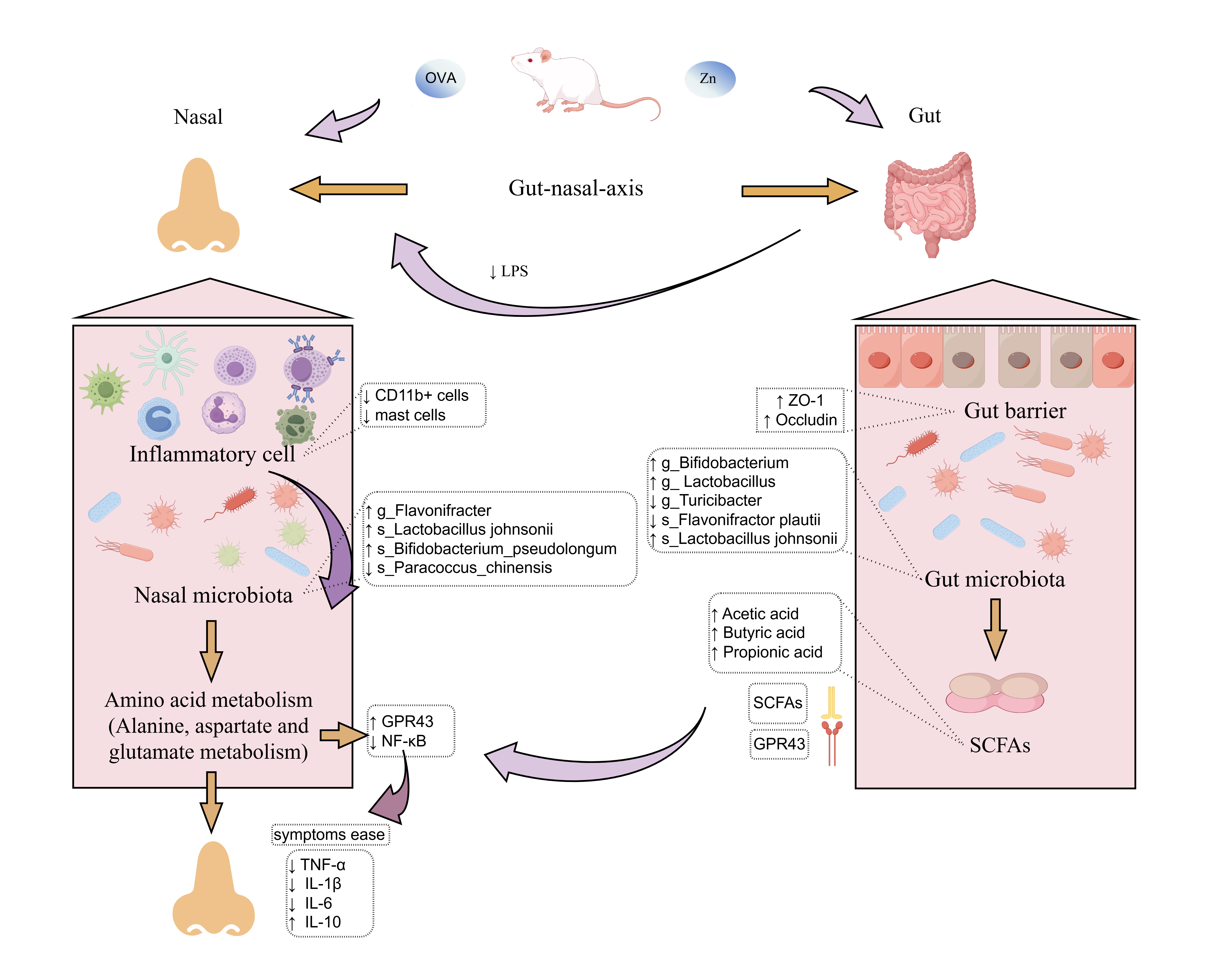

Supplement: Supplementary file 2 [file Image2.tif]
